# 

**Published:** 1862-07

**Authors:** 


### CONTENTS.

### ORIGINAL CONTRIBUTIONS.

ART. XIX. Sanitary Condition and Wants of Chicago. By N. S. Davis, M.D. & Prof. .... 395
ART. XX. Fatty Degeneration of the Kidneys. Cardiac Hypertrophy. Death. Autopsy. By James P. Ross, M.D. .... 405
ART. XXI. Strychnia in Habitual Constipation. By E. H. Neyman, M.D., Cedar Bluffs, Iowa .... 409
ART. XXII, Chronic Orchitis, and Extirpation of Testicle. By L. D. Robinson, M.D., Groveland, Ind. .... 414
ART. XXIII. Case of extensive Injury of the Head. By H. Wanzer, M.D. .... 418

### ARMY CORRESPONDENCE.

Reply to a Slanderous Attack, published in the Chicago Medical Journal, May number. By R. Roskoten .... 421

### SELECTIONS.

Epilepsy. After the French of Herpin. By Edward Sutton Smith, M.D. .... 431
The Sarracenia Purpurea, a Remedy for Small-Pox .... 438
Oiled Paper, as an Economical Substitute for Oiled Silk in Surgical Dressings .... 440
Chloride of Lime as an Insecticide .... 441

### BOOK NOTICES.

American Journal of Opthalmology. Vol., No. 1, By Julius Homberger, M.D., Editor and Proprietor. New York .... 441
A Practical guide to the Study of the Diseases of the Eye: Their Medical and Surgical Treatment. By Henry W. Williams, M.D., etc. 442
On Military and Camp Hospitals, and the Health of Troops in the Field. By L. Bandens, M.D., etc .... 442
The Domain of Medical Police. By Louis Elsberg, M.A., M.D. .... 443
Advice to a Mother on the Management of her Offspring. By Pye H. Chevasse, M.D., etc .... 444
London Lancet .... 444

### EDITORIAL.

Army Surgeons .... 444
Clinical Cases in the Medical Wards of the Mercy Hospital.
Organic Desease of the Heart.—Anasarca, &c .... 446
Syphilitic Ecthyma .... 447
Chronic Diarrhœa.—Ruebola, &c .... 449
Medical Department Lind University .... 451
Army Assistant-Surgeons .... 452
Self-Applying Sponge Pessary .... 452
The Treatment of Aneurism .... 454
The Poison of the Adder .... 455
Acetate of Potash in Gonorrhœa .... 455
A Human Salamander .... 455
Ophthalmia .... 456
The Hairless Men of Australia .... 456
Test for Poisonous Paper-Hangings .... 456
Grand Monarch, Louis XIV .... 456
Medical News .... 457

